# Supplementary material for: Discovery of VIP236, an αvβ3-Targeted Small-Molecule–Drug Conjugate with Neutrophil Elastase-Mediated Activation of 7-Ethyl Camptothecin Payload for Treatment of Solid Tumors
Source: Cancers (Basel). 2023 Sep 1;15(17):4381. doi: 10.3390/cancers15174381 (PMC10486604; doi:10.3390/cancers15174381)
Supplement: Supplementary file 1 [file cancers-15-04381-s001.zip › cancers-2509186-supplementary.pdf]

Discovery of VIP236, an αvβ3-Targeted Small Molecule–Drug Conjugate with Neutrophil Elastase-Mediated Activation of 7-Ethyl Camptothecin Payload for Treatment of Solid Tumors

Hans-Georg Lerchen[1*], Beatrix Stelte-Ludwig[1], Melanie Heroult[2], Dmitry Zubov[3], Kersten Matthias Gericke[3], Harvey Wong[4], Melanie M. Frigault[4], Amy J. Johnson[4], Raquel Izumi[4], Ahmed Hamdy[4]

Supplementary data **1**
1. Synthesis of SMDCs 1
General Procedures 1
Synthesis of 8 (VIP236) 2
a) Synthesis of activated integrin ligand **7** 2
b) Synthesis of linker peptide **4** 3
c) Synthesis of the SMDC **8** 4
Synthesis of the non-cleavable SMDC epimer 8e1 15
Synthesis of weakly binding epimer 8e2 17
a) Synthesis of activated integrin ligand epimer **7e2** 17
b) Synthesis of the weakly binding SMDC epimer **8e2** 18
2. Supplementary tables and figures 20
a) Supplementary tables 20
b) Supplementary Figures 21

Supplementary data

1. Synthesis of SMDCs

General Procedures

All commercial reagents and catalysts were used as provided by the commercial supplier without purification. Solvents for synthesis, extraction and chromatography were of reagent grade and used as received. Moisture-sensitive reactions were carried out under an atmosphere of argon, and anhydrous solvents were used as provided by the commercial supplier.

1H NMR and 13C NMR spectra were recorded at room temperature (RT) with Bruker Avance spectrometers. Chemical shifts (δ) are reported in ppm relative to TMS as an internal standard.

The descriptions of the coupling patterns of 1H NMR signals are based on the optical appearance of the signals and do not necessarily reflect the physically correct interpretation. In general, the chemical shift information refers to the center of the signal. In the case of multiplets, intervals are given.

Analytical mass spectrometry was performed on HPLC/MS (Waters, Agilent, Thermo Fisher) using Waters Time-of-Flight, Waters/Micromass Single Quadrupole, or Thermo Fisher Scientific Orbitrap mass spectrometers. Ionization methods were electrospray ionization (ESI) positive/negative or electron ionization (EI).

LC/MS analyses were performed using the respective method as noted.

Method 1:

Instrument: Waters ACQUITY SQD UPLC System; Column: Waters Acquity UPLC HSS T3 1.8 µ 50 x 1 mm; Eluent A: 1 l Water + 0.25 mL 99% formic acid, Eluent B: 1 l acetonitrile + 0.25 mL 99% formic acid; Gradient: 0.0 min 90% A → 1.2 min 5% A → 2.0 min 5% A Stove: 50°C; Flow: 0.40 mL/min; UV-Detection: 208 – 400 nm.

Method 2.

Instrument: Thermo Scientific FT-MS with Thermo Scientific UltiMate 3000 UHPLC; column: Waters HSS T3 C18 1.8 µm, 75 mm × 2.1 mm; eluent A: water + 0.01% formic acid; eluent B: acetonitrile + 0.01% formic acid; gradient: 0.0 min 10% B → 2.5 min 95% B → 3.5 min 95% B; temperature: 50 °C; flow rate: 0.90 mL/min; UV detection: 210–400 nm.

Method 3.

System MS: Waters TOF instrument; System UPLC: Waters Acquity I-CLASS; Column: Waters Acquity UPLC HSS T3 1.8 µm 50 x 1 mm; eluent A: water + 0.01% formic acid, eluent B: acetonitrile + 0.01% formic acid; gradient: 0.0 min 95% A → 6.0 min 5% A → 7.5 min 5% A, temperature: 50°C; flow rate: 0.35 mL/min; UV-Detection: 210 nm.

Synthesis of 8 (VIP236)

a) *Synthesis of activated integrin ligand* **7**

(3R)-3-{[(4-{[(4-nitrophenoxy)carbonyl]amino}phenyl)carbamoyl]amino}-3-{3-[({3-[(propyl carbamoyl)amino]phenyl}sulfonyl)amino]phenyl}propanoic acid (**7 R enantiomer)**

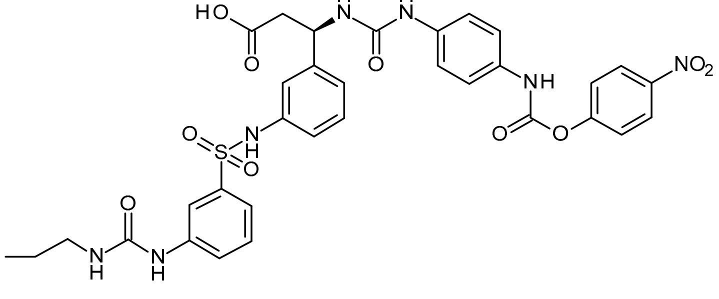

The synthesis of has been described in patent WO2020/094471 [40].

LC/MS (Method 1): Rt = 0.97 min; MS (ESIpos): m/z = 720 $(M+H)^+$.

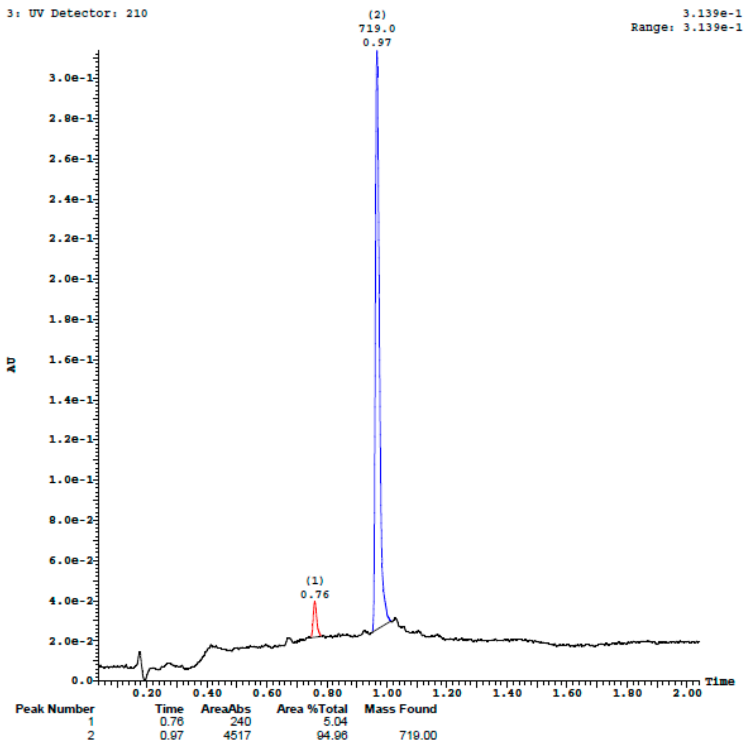

Chiral HPLC: For compound **7** an ee > 99% has been determined by chiral supercritical fluid chromatography (SFC). The results are shown in Table S1.

Column: Daicel Chiralpak ID-3 3µm, 100 x 4,6 mm, Temp.: 40°C

Eluent: 60% CO_2: 40% ETOH

Flow: 3.0 mL/min, Run time regular: 10min, UV: 210nm, Inj.: 5 µl

SFC-BPR Pressure: 130 bar, SFC-BPR Temp.: 60°C

b) *Synthesis of linker peptide **4***

The synthesis of the linker peptide (2S)-1-[(19S)-19-(2-tert-butoxy-2-oxoethyl)-2,2-dimethyl-4,17,20-trioxo-3,8,11,14-tetraoxa-5,18-diazaicosan-20-yl]pyrrolidine-2-carboxylic acid **4** has been described in WO2020/094471.

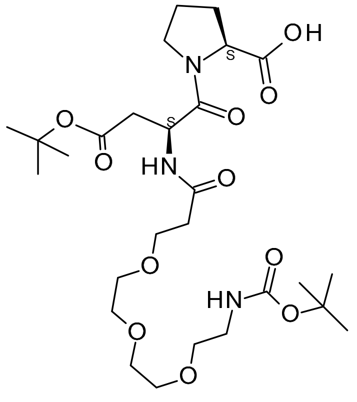

LC/MS (Method 1): Rt = 0.86 min; MS (ESIpos): m/z = 590 (M+H)+.

c) *Synthesis of the SMDC **8***

Disodium (4S)-4,11-diethyl-3,14-dioxo-3,4,12,14-tetrahydro-1H-pyrano[3',4':6,7]indolizino [1,2-b]quinoline-4-yl 1-{(2S)-2-(carboxylatomethyl)-17-[4-({[(1R)-2-carboxylato-1-{3-[({3-[(propylcarbamoyl)amino]phenyl}sulfonyl)amino]phenyl}ethyl]carbamoyl} amino)anilino]-4,17-dioxo-7,10,13-trioxa-3,16-diazaheptadecan-1-oyl}-L-prolyl-L-valinate

The synthesis of **8** has been up-scaled following Scheme S1 as described below:

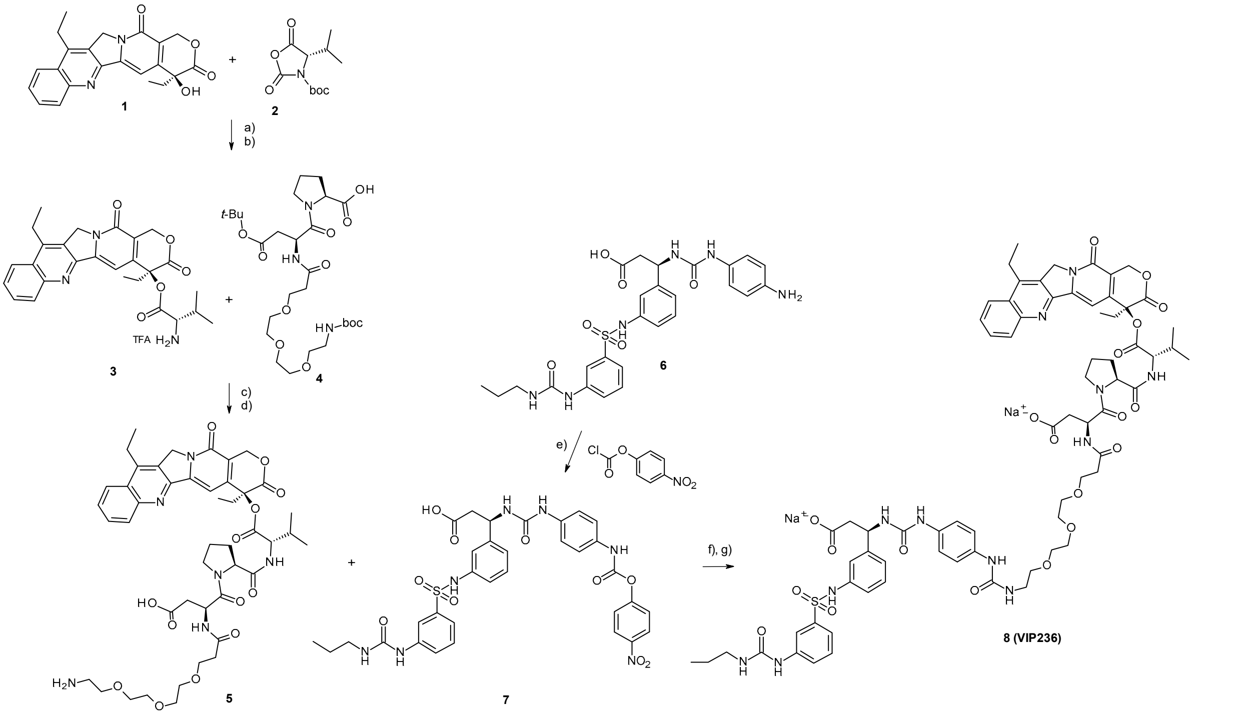

Scheme S1: Synthesis of the SMDC **8** (VIP236)

(4*S*)-4,11-diethyl-3,14-dioxo-3,4,12,14-tetrahydro-1H-pyrano[3',4':6,7]indolizino[1,2-b]quinolin-4-yl N-(tert-butoxycarbonyl)-L-valinate (3-boc)

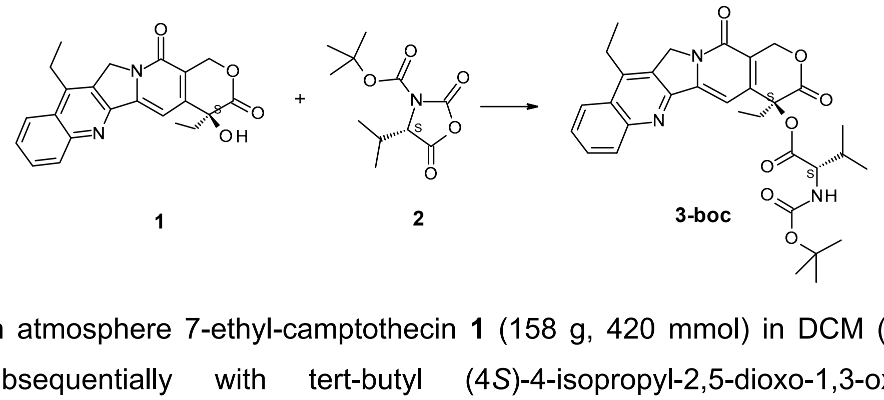

Under argon atmosphere 7-ethyl-camptothecin **1** (158 g, 420 mmol) in DCM (5.50 L) was treated subsequentially with tert-butyl (4*S*)-4-isopropyl-2,5-dioxo-1,3-oxazolidine-3-carboxylate **2** (93% purity, 220 g, 840 mmol, 2.0 equiv.) and DMAP (38.5 g, 315 mmol, 0.75 equiv.) at RT. The suspension stirred under reflux overnight. The reaction solution was washed

with 5% aq. citric acid (2 x 1.50 L) and water (1.00 L). The organic phase was dried over $MgSO_4$, filtered, and concentrated under reduced pressure. The residue was stirred in acetonitrile/diethyl ether (2.50 L, 1:1) for 1 h. The solid was filtered off, washed with diethyl ether (2 x 300 mL) and dried to give 216 g (4*S*)-4,11-diethyl-3,14-dioxo-3,4,12,14-tetrahydro-1H-pyrano[3',4':6,7]indolizino[1,2-b]quinolin-4-yl N-(tert-butoxycarbonyl)-L-valinate (**3-boc**) (yield: 89%).

LC/MS (Method 2): R_t = 2.27 min, m/z = 576.3 ($M+H^+$)

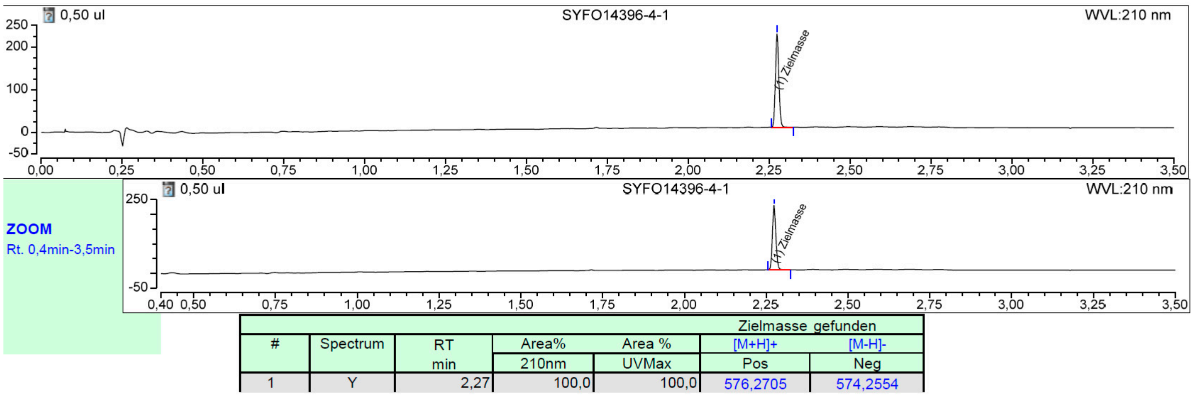

#	Spectrum	RT	Area%	Area %	Zielmasse gefunden [M+H]+	[M-H]-
		min	210nm	UVMax	Pos	Neg
1	Y	2,27	100,0	100,0	576,2705	574,2554

^{1}H NMR (600 MHz, DMSO-d_6) δ ppm 8.29 (d, *J* = 8.6 Hz, 1 H), 8.10 (br d, *J* = 8.4 Hz, 1 H), 7.86 (t, *J* = 7.5 Hz, 1 H), 7.73 (t, *J* = 7.2 Hz, 1 H), 7.43 (br d, *J* = 7.8 Hz, 1 H), 7.27 (s, 1 H), 5.50 (s, 2 H), 5.28 - 5.41 (m, 2 H), 3.90 (br t, *J* = 7.2 Hz, 1 H), 3.19 - 3.26 (m, 2 H), 2.14 (dq, *J* = 12.3, 6.3 Hz, 3 H), 1.52 (s, 9 H), 1.31 (t, *J* = 7.5 Hz, 3 H), 0.88 - 0.99 (m, 9 H).

The NMR spectrum shows a few additional signals (not listed above) which are caused by rotamers (see spectra below).

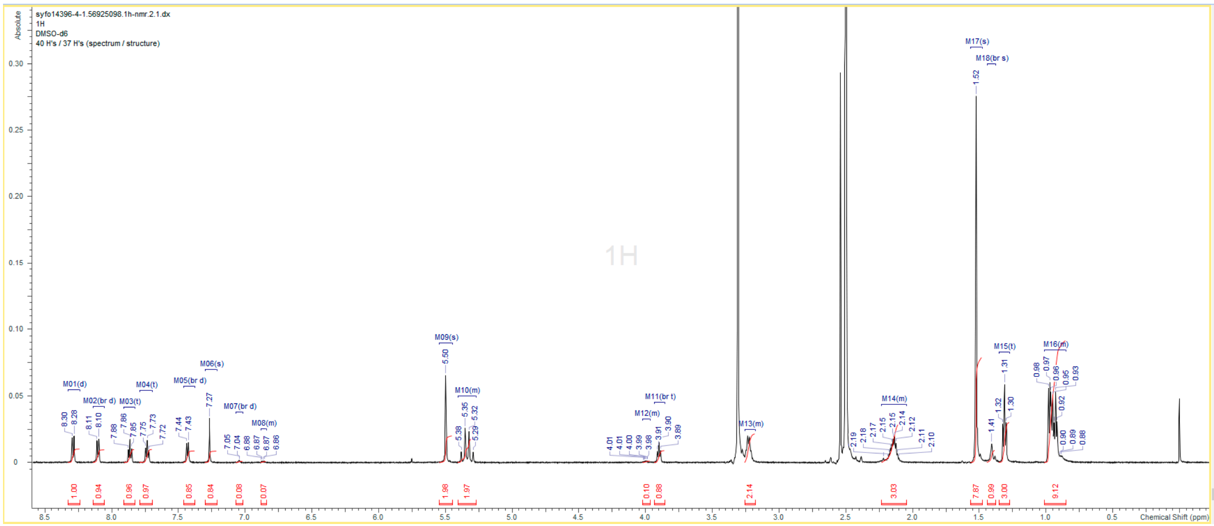

(4*S*)-4,11-diethyl-3,14-dioxo-3,4,12,14-tetrahydro-1H-pyrano[3',4':6,7]indolizino[1,2-b]quinolin-4-yl L-valinate tri-fluoroacetate (3):

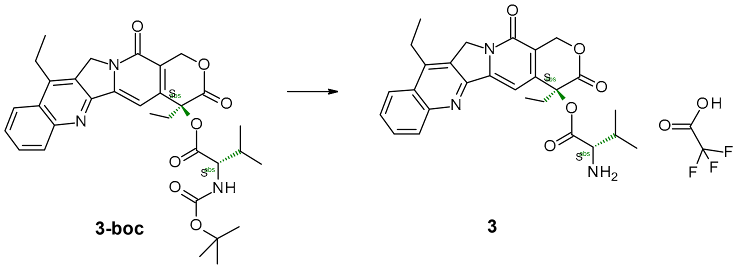

To a solution of (4*S*)-4,11-diethyl-3,14-dioxo-3,4,12,14-tetrahydro-1H-pyrano[3',4':6,7] indolizino[1,2-b]quinolin-4-yl N-(tert-butoxycarbonyl)-L-valinate **3-boc** (276 g, 479 mmol) in DCM (2.0 L) TFA (400 mL) was added at RT and stirring was continued for 3 h. The solution was concentrated under reduced pressure and for TFA removal the residue was co-evaporate with toluene (2 x 400 mL). The residue was dissolved in DCM/MeOH (1.1 L, 10:1) and then added dropwise into diisopropyl ether (8.0 L). The suspension was stirred for 1h, filtered and the solid was washed with diisopropyl ether (2 x 200 mL) and dried in the fume hood overnight to give 300 g (4*S*)-4,11-diethyl-3,14-dioxo-3,4,12,14-tetrahydro-1H-pyrano[3',4':6,7]indolizino [1,2-b]quinolin-4-yl L-valinate tri-fluoroacetate (**3**) (yield: quantitative, TFA content: 18.1%).

LC/MS (Method 3): R_t = 1.98 min, m/z = 476.2 ($M+H^+$)

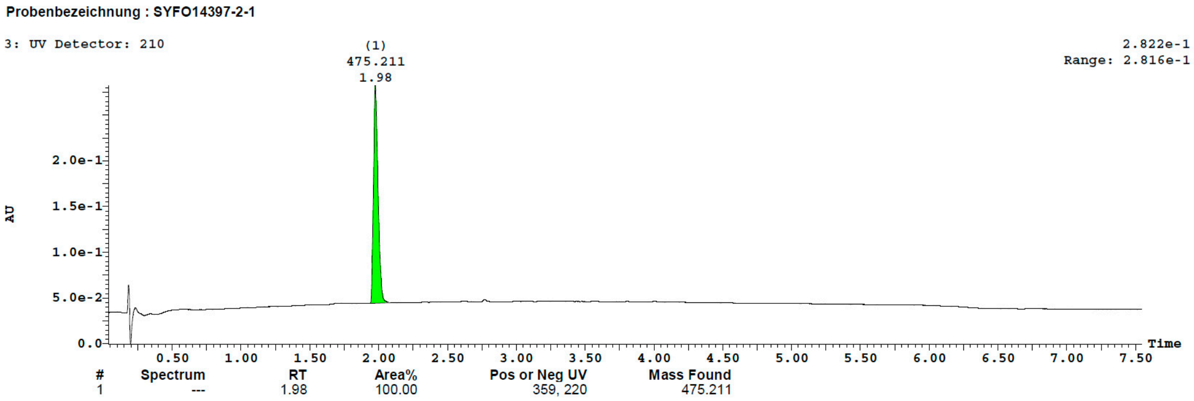

^{1}H NMR (600 MHz, DMSO-d_6) δ ppm 8.51 (br s, 3 H), 8.32 (d, *J*=8.4 Hz, 1 H), 8.15 (d, *J*=8.4 Hz, 1 H), 7.87 (t, *J*=7.6 Hz, 1 H), 7.76 (t, *J*=7.6 Hz, 1 H), 7.32 (s, 1 H), 5.47 - 5.62 (m, 2 H), 5.32 - 5.43 (m, 2 H), 4.29 (br d, *J*=1.2 Hz, 1 H), 3.19 - 3.27 (m, 3 H), 2.20 - 2.31 (m, 2 H), 1.32 (t, *J*=7.4 Hz, 3 H), 1.10 (br d, *J*=6.8 Hz, 3 H), 1.04 (d, *J*=6.8 Hz, 4 H), 0.96 (br t, *J*=7.3 Hz, 3 H).

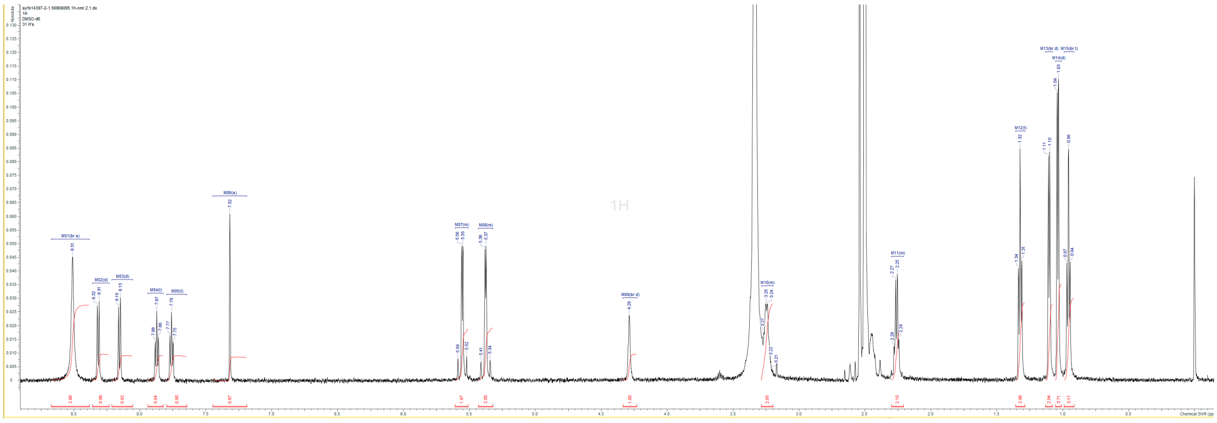

tert-Butyl (19*S*)-19-{[(2*S*)-2-{[(2*S*)-1-{[(4*S*)-4,11-diethyl-3,14-dioxo-3,4,12,14-tetrahydro-1H-pyrano[3',4':6,7]indo-lizino[1,2-b]quinolin-4-yl]oxy}-3-methyl-1-oxobutan-2-yl]carbamoyl}pyrrolidin-1-yl]carbonyl}-2,2-dimethyl-4,17-dioxo-3,8,11,14-tetraoxa-5,18-diazahenicosan-21-oate (5-boc):

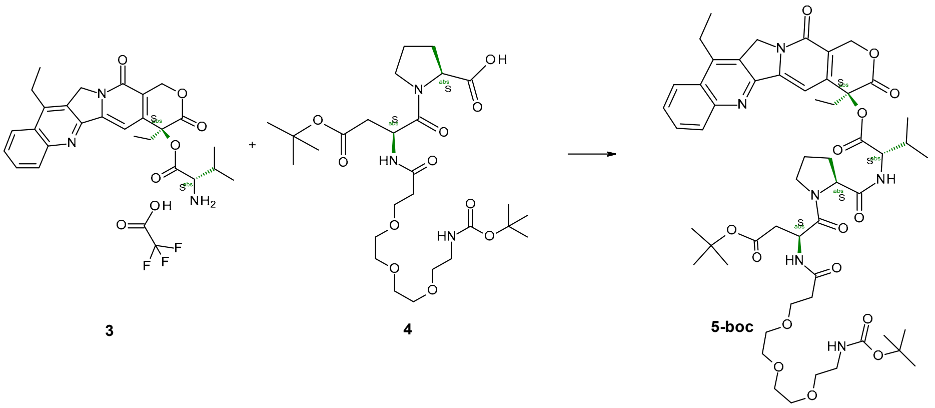

3 **4** **5-boc**

Under argon atmosphere (2*S*)-1-[(19*S*)-19-(2-tert-butoxy-2-oxoethyl)-2,2-dimethyl-4,17,20-trioxo-3,8,11,14-tetraoxa-5,18-diazaicosan-20-yl]pyrrolidine-2-carboxylic acid **4** (137 g, 232 mmol, 1.05 equiv.) was dissolved in DMF (2.5 L) and EDCI (46.6 g, 243 mmol, 1.1 equiv.) and oxyma® (40.8 g, 287 mmol, 1.3 equiv.) were added at 0 °C. After addition of (4*S*)-4,11-diethyl-3,14-dioxo-3,4,12,14-tetrahydro-1H-pyrano[3',4':6,7]indolizino[1,2-b]quinolin-4-yl L-valinate tri-fluoroacetate **4** (130 g, 221 mmol) DIPEA (115 mL, 85.6 g, 662 mmol, 3.0 equiv.) was added dropwise. The reaction mixture was stirred overnight at 0 °C. The reaction mixture was diluted with EtOAc (8.0 L) and then washed with 10% aq. citric acid (2 x 4.0 L), 10% aq. sodium hydrogen carbonate in water (2 x 4.0 L) and satd. aq. sodium chloride (2 x 3.0 L). The organic layer was dried over $MgSO_4$, filtered, and concentrated under reduced pressure. The residue was dissolved in DCM (700 mL) and purified flash chromatography (Biotage Isolera LS system, 800 g Biotage KP-NH silica gel, DCM/MeOH 100:1, 6.0 L). The product containing fractions were concentrated under reduced pressure to give 204 g tert-butyl (19*S*)-19-{[(2*S*)-2-{[(2*S*)-1-{[(4*S*)-4,11-diethyl-3,14-dioxo-3,4,12,14-tetrahydro-1H-pyrano[3',4':6,7]indo-lizino[1,2-

b]quinolin-4-yl]oxy}-3-methyl-1-oxobutan-2-yl]carbamoyl}pyrrolidin-1-yl]carbonyl}-2,2-dimethyl-4,17-dioxo-3,8,11,14-tetraoxa-5,18-diazahenicosan-21-oate (**5-boc**) (yield: 84%).

LC/MS (Method 3): R_t = 3.86 min, m/z = 1047.5 ($M+H^+$)

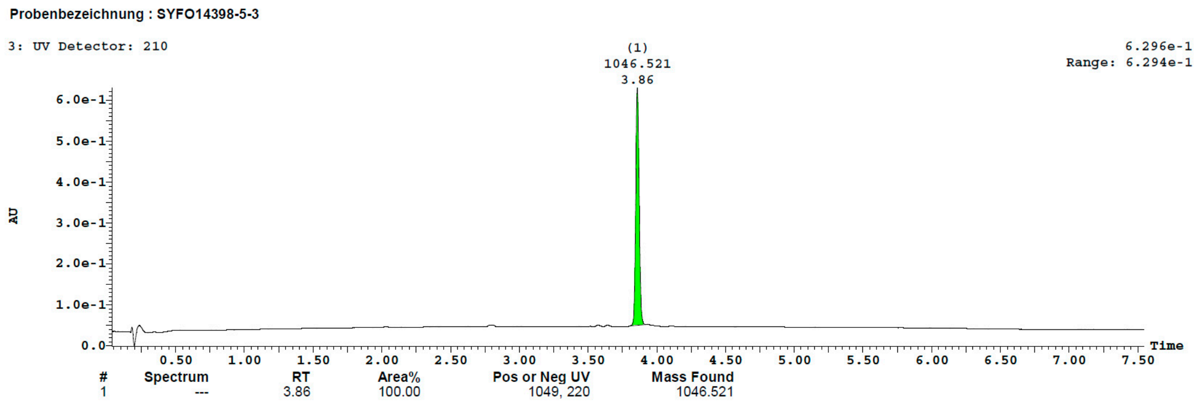

^{1}H NMR (600 MHz, DMSO-d_6) δ ppm 8.34 (br d, J = 8.4 Hz, 1 H), 8.28 (br t, J = 6.9 Hz, 2 H), 8.08 (br d, J = 8.4 Hz, 1 H), 7.86 (br t, J = 7.6 Hz, 1 H), 7.74 (br t, J = 7.5 Hz, 1 H), 7.15 (s, 1 H), 6.72 (br s, 1 H), 5.44 - 5.55 (m, 2 H), 5.26 - 5.40 (m, 2 H), 4.87 - 4.98 (m, 1 H), 4.71 (br d, J = 8.6 Hz, 1 H), 4.18 (br t, J = 7.9 Hz, 1 H), 3.56 - 3.72 (m, 4 H), 3.42 - 3.52 (m, 8 H), 3.36 (br t, J = 6.0 Hz, 2 H), 3.22 (q, J = 6.9 Hz, 2 H), 3.05 (q, J = 5.5 Hz, 2 H), 2.64 (br dd, J = 16.0, 7.4 Hz, 1 H), 2.31 - 2.40 (m, 3 H), 2.10 - 2.26 (m, 4 H), 1.82 - 2.05 (m, 3 H), 1.28 - 1.41 (m, 21 H), 0.89 - 0.99 (m, 9 H).

The NMR spectrum shows a few additional signals (not listed above) which are caused by rotamers and as well traces of DCM and DMF (see spectra below).

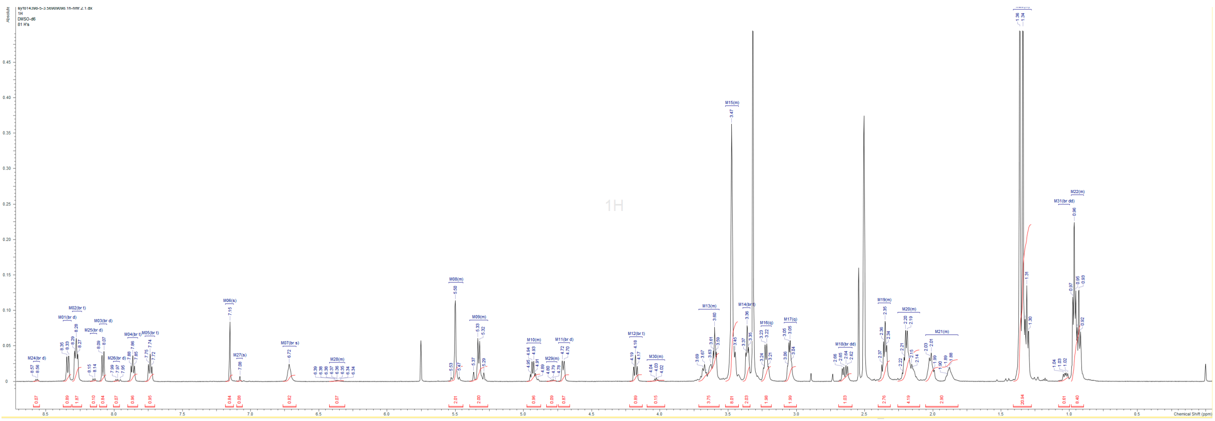

(4*S*)-4,11-Diethyl-3,14-dioxo-3,4,12,14-tetrahydro-1H-pyrano[3',4':6,7]indolizino[1,2-b]quinolin-4-yl N-(3-{2-[2-(2-aminoethoxy)ethoxy]ethoxy}propanoyl)-L-alpha-aspartyl-L-prolyl-L-valinate trifluoroacetate (5):

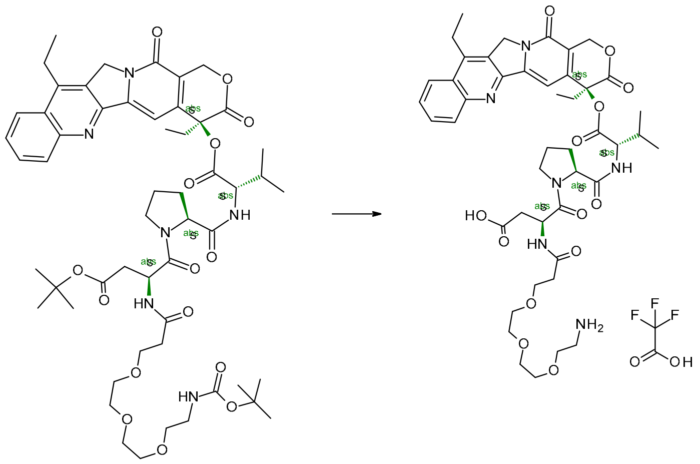

To a solution of tert-butyl (19*S*)-19-{[(2S)-2-{[(2*S*)-1-{[(4*S*)-4,11-diethyl-3,14-dioxo-3,4,12,14-tetrahydro-1H-pyrano[3',4':6,7]indolizino[1,2-b]quinolin-4-yl]oxy}-3-methyl-1-oxobutan-2-yl]carbamoyl}pyrrolidin-1-yl]carbonyl}-2,2-dimethyl-4,17-dioxo-3,8,11,14-tetraoxa-5,18-diazahenicosan-21-oate **5-boc** (104 g, 99.3 g) in DCM (1.00 L) TFA (1.00 L, 13.0 mol) was added and stirring was continued at RT for 23 h. LC/MS indicated complete reaction. The reaction mixture was concentrated under reduced pressure and then co-evaporated with toluene (500 mL) for removal of excess TFA. The obtained solid was dissolved in DCM/MeOH (100 mL, 10:1) and then added dropwise under stirring to diethyl ether (2.5 L). The suspension was stirred overnight, filtered and the solid was washed with diethyl ether (2 x 150 mL). The bright yellow solid was dried under reduced pressure to give 110 g (4*S*)-4,11-Diethyl-3,14-dioxo-3,4,12,14-tetrahydro-1H-pyrano[3',4':6,7]indolizino[1,2-b]quinolin-4-yl N-(3-{2-[2-(2-aminoethoxy)ethoxy]ethoxy}propanoyl)-L-alpha-aspartyl-L-prolyl-L-valinate trifluoroacetate (**5**) (yield: quantitative, TFA content: 14.3%).

LC/MS (Method 3): R_t = 0.73 min, m/z = 891.4 (M+H$^+$)

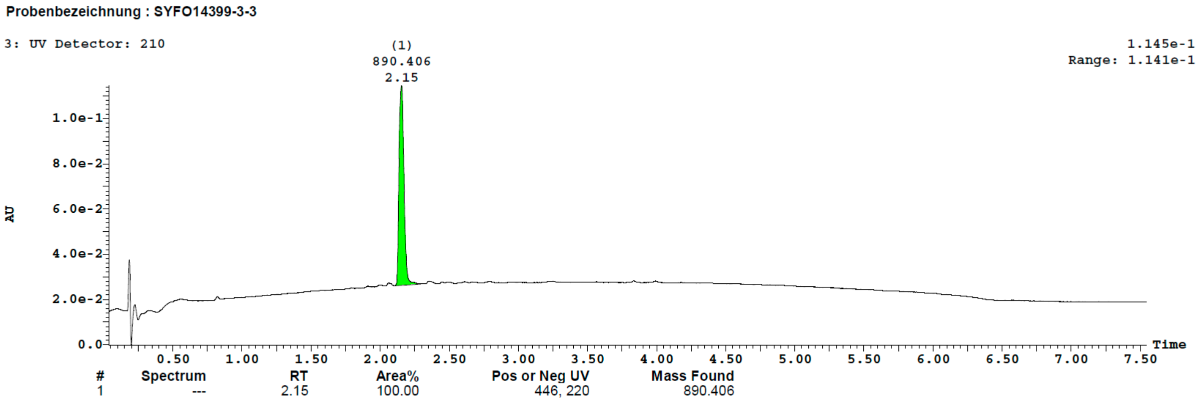

^{1}H NMR (600 MHz, DMSO-d_6) δ ppm 12.2 - 12.5 (m, 1 H), 8.36 (d, *J* = 8.0 Hz, 1 H), 8.30 (d, *J* = 8.2 Hz, 1 H), 8.10 - 8.17 (m, 1 H), 8.06 (d, *J* = 8.4 Hz, 1 H), 7.86 (t, *J* = 7.6 Hz, 1 H), 7.63 - 7.80 (m, 4 H), 7.22 (s, 1 H), 5.49 (s, 2 H), 5.29 - 5.40 (m, 2 H), 4.91 (q, *J* = 7.2 Hz, 1 H), 4.80

(dd, J = 8.3, 2.8 Hz, 1 H), 4.12 (t, J = 8.0 Hz, 1 H), 3.33 - 3.78 (m, 40 H), 3.23 (q, J = 7.4 Hz, 3 H), 2.90 - 3.03 (m, 2 H), 2.74 (dd, J = 16.7, 6.6 Hz, 1 H), 2.09 - 2.47 (m, 9 H), 1.86 - 2.07 (m, 3 H), 1.31 (t, J = 7.6 Hz, 3 H), 1.09 (t, J = 7.0 Hz, 3 H), 0.87 - 1.04 (m, 9 H).

Some signals in the NMR spectrum are covered by a water peak which leads to an insufficient integration (see spectra below).

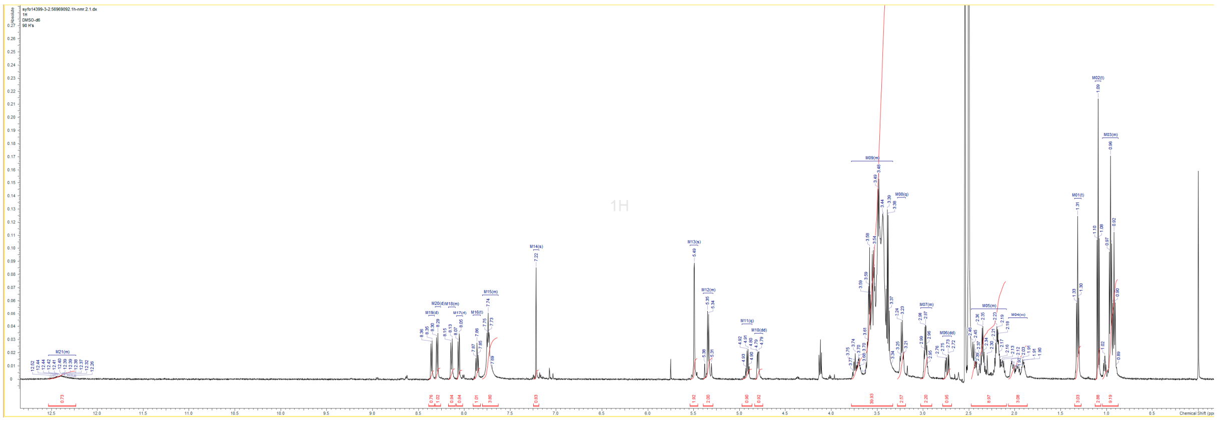

(4*S*)-4,11-Diethyl-3,14-dioxo-3,4,12,14-tetrahydro-1H-pyrano[3',4':6,7]indolizino[1,2-b]quinolin-4-yl N-{14-[4-({[(1*R*)-2-carboxy-1-{3-[({3-[(propylcarbamoyl)amino]phenyl} sulfonyl)amino]phenyl}ethyl]carbamoyl}amino) anilino]-14-oxo-4,7,10-trioxa-13-azatetra-decan-1-oyl}-L-alpha-aspartyl-L-prolyl-L-valinate (8 free acid):

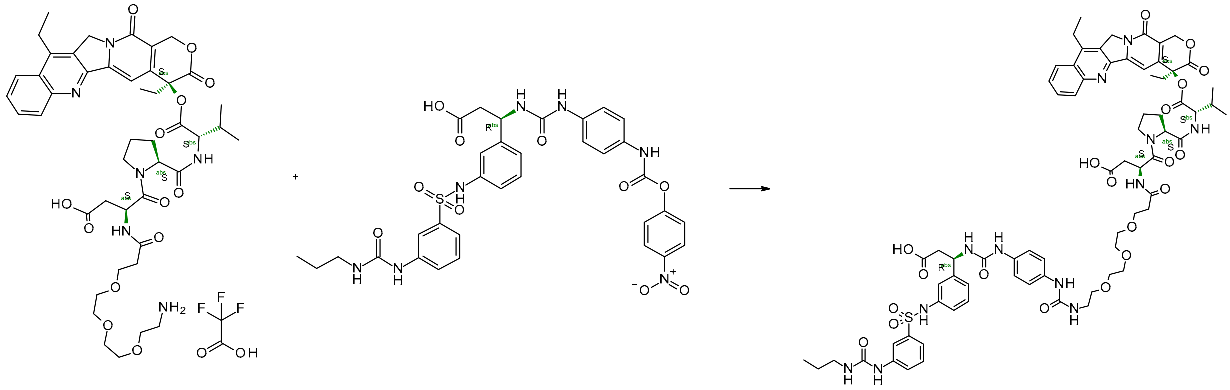

5 **7** **8 (free acid)**

To a solution of (4S)-4,11-diethyl-3,14-dioxo-3,4,12,14-tetrahydro-1H-pyrano[3',4':6,7] indolizino[1,2-b]quinolin-4-yl-N-(3-{2-[2-(2-aminoethoxy)ethoxy]ethoxy}propanoyl)-L-alpha-aspartyl-L-prolyl-L-valinate trifluoroacetate **5** (8.75 g, 8.53 mmol) and (3*R*)-3-{[(4-{[(4-nitrophenoxy)carbonyl]amino}phenyl) carbamoyl]amino}-3-{3-[({3-[(propylcarbamoyl) amino] phenyl} sulfonyl)amino]phenyl}propanoic acid (6.20 g, 8.53 mmol, 1.0 equiv.) in DMF (46 mL) were added DIPEA (14.9 mL, 11.0 g, 85.3 mmol, 10 equiv.) at RT The reaction mixture was stirred for 3 h at RT The reaction mixture was treated with 10% aq. citric acid (500 mL) and

stirred for 10 min. The suspension was filtered, and the residue was washed with water (2 x 250 mL). The solid was dried overnight under reduced pressure to give 12.1 g crude product. This material was purified by SFC chromatography to give 8.86 g (4*S*)-4,11-diethyl-3,14-dioxo-3,4,12,14-tetrahydro-1H-pyrano[3',4':6,7]indolizino[1,2-b]quinolin-4-yl N-{14-[4-({[(1*R*)-2-carboxy-1-{3-[({3-[(propylcarbamoyl)amino]phenyl} sulfonyl)amino]phenyl}ethyl]carbamoyl}amino) anilino]-14-oxo-4,7,10-trioxa-13-azatetra-decan-1-oyl}-L-alpha-aspartyl-L-prolyl-L-valinate (**8 free acid**) (yield: 71%).

SFC chromatography: Sample preparation: 12.1 g crude material dissolved in 710 mL methanol and 710 mL acetonitrile and filtered; SFC: Prep Novasep; column: Dr. Maisch Reprosphere 2-ethyl pyridine, 10 µm, 360 x 50 mm (achiral); eluent: CO_2 50% / methanol 50% isocratic; injection volume: 15 mL (~137 mg), flow rate: 300 mL/min; total runtime: 18 min; UV: 210 nm; backpressure 110 bar; temperature: 40 °C.

LC/MS (Method 3): R_t = 2.97 min, m/z = 1471.6 ($M+H^+$).

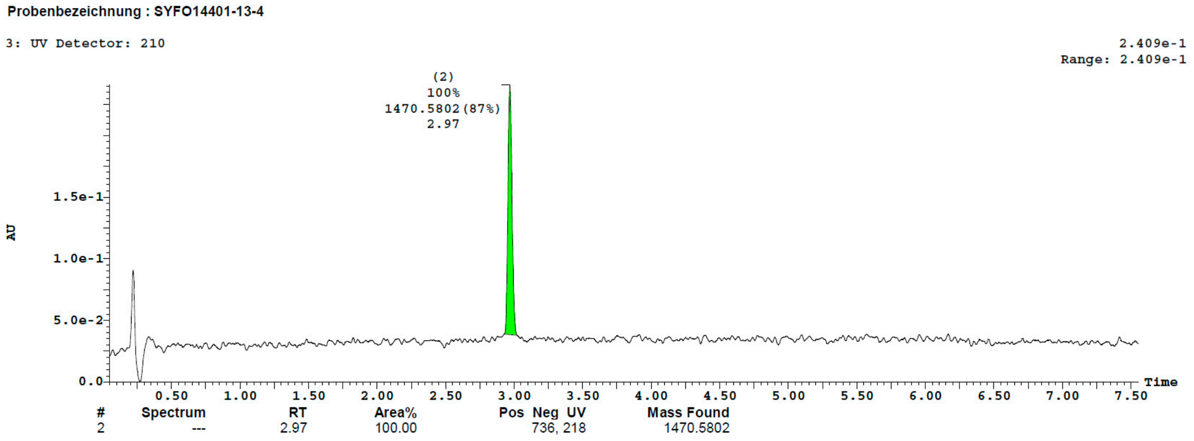

^{1}H NMR (600 MHz, DMSO-d_6) δ ppm 12.4 (br s, 2 H), 10.3 (s, 1 H), 8.78 (br s, 1 H), 8.30 - 8.44 (m, 3 H), 8.26 (br d, *J* = 8.4 Hz, 1 H), 8.15 (br d, *J* = 8.4 Hz, 1 H), 8.01 - 8.09 (m, 2 H), 7.86 (br t, *J* = 7.5 Hz, 1 H), 7.72 (br t, *J* = 7.4 Hz, 1 H), 7.42 (br d, *J* = 7.6 Hz, 1 H), 7.12 - 7.34 (m, 9 H), 7.00 (br d, *J* = 7.8 Hz, 1 H), 6.91 (br d, *J* = 8.0 Hz, 1 H), 6.64 (br d, *J* = 8.2 Hz, 1 H), 6.23 (br s, 1 H), 6.06 (br t, *J* = 5.3 Hz, 1 H), 5.50 (s, 2 H), 5.24 - 5.38 (m, 2 H), 5.01 (q, *J* = 7.2 Hz, 1 H), 4.91 - 4.97 (m, 1 H), 4.86 (dd, *J* = 8.3, 2.6 Hz, 1 H), 4.10 (t, *J* = 8.0 Hz, 1 H), 3.67 - 3.85 (m, 2 H), 3.60 (t, *J* = 6.5 Hz, 2 H), 3.48 (br d, *J* = 2.9 Hz, 7 H), 3.42 (br t, *J* = 5.5 Hz, 2 H), 3.35 (br s, 2 H), 3.15 - 3.27 (m, 4 H), 3.04 (q, *J* = 6.5 Hz, 2 H), 2.76 (dd, *J* = 16.7, 6.8 Hz, 1 H), 2.65 (br d, *J* = 6.9 Hz, 2 H), 2.46 (br dd, *J* = 16.7, 6.9 Hz, 1 H), 2.31 - 2.39 (m, 2 H), 2.11 - 2.27 (m, 4 H), 1.88 - 2.06 (m, 3 H), 1.39 - 1.48 (m, 2 H), 1.30 (br t, *J* = 7.5 Hz, 3 H), 0.95 - 1.04 (m, 6 H), 0.93 (t, *J* = 7.4 Hz, 3 H), 0.86 (t, *J* = 7.4 Hz, 3 H).

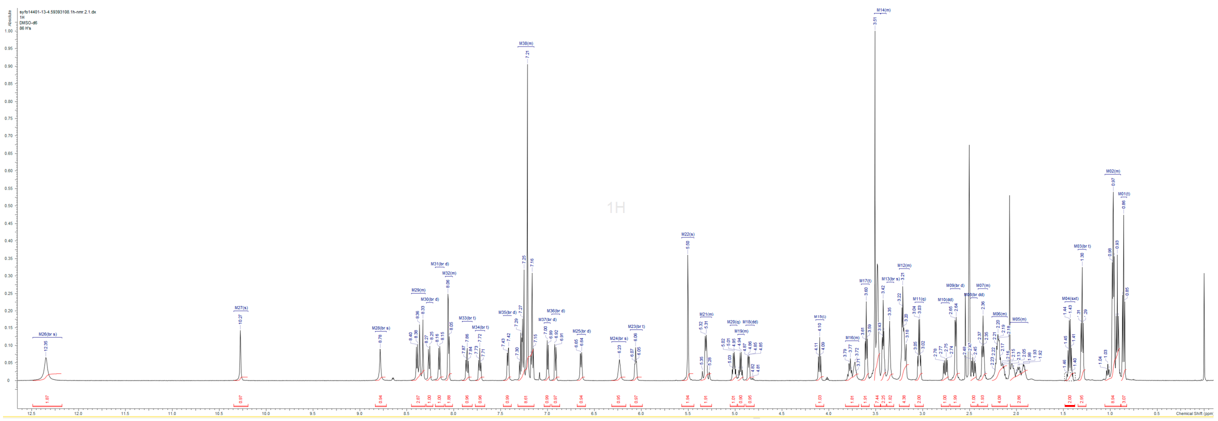

Disodium (4*S*)-4,11-diethyl-3,14-dioxo-3,4,12,14-tetrahydro-1H-pyrano[3',4':6,7] indolizino[1,2-b]quinolin-4-yl 1-{(2*S*)-2-(carboxylatomethyl)-17-[4-({[(1*R*)-2-carboxylato-1-{3-[({3-[(propylcarbamoyl)amino]phenyl}sulfonyl)amino]phenyl}ethyl]carbamoyl} amino)anilino]-4,17-dioxo-7,10,13-trioxa-3,16-diazaheptadecan-1-oyl}-L-prolyl-L-valinate (8):

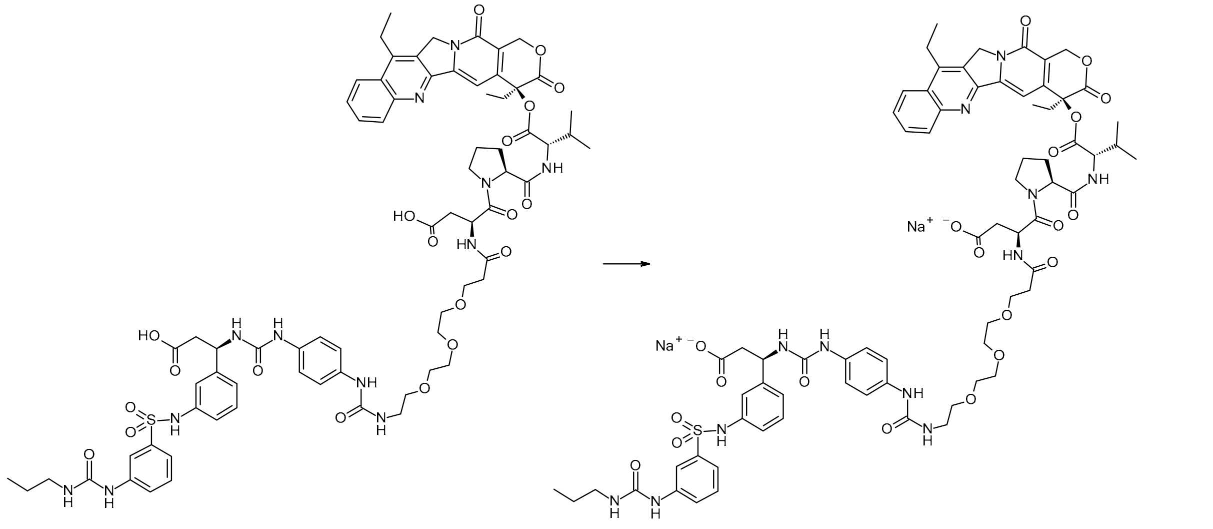

8 free acid **8**

(4S)-4,11-diethyl-3,14-dioxo-3,4,12,14-tetrahydro-1H-pyrano[3',4':6,7]indolizino[1,2-b] quinoline-4-yl N-{14-[4-({[(1R)-2-carboxy-1-{3-[({3-[(propylcarbamoyl)amino]phenyl} sulfonyl) amino]phenyl}ethyl]carbamoyl}amino) anilino]-14-oxo-4,7,10-trioxa-13-azatetra-decan-1-oyl}-L-alpha-aspartyl-L-prolyl-L-valinate **8 free acid** (46.8 g, 31.8 mmol) was dissolved in acetone (500 ml) and water (50 mL) under ultra-sonification for 20 min. Sodium bicarbonate (5.35 g, 63.6 mmol, 2.0 equiv.) in 150 mL water was added and stirring was continued for 1 h. Afterwards, acetone was removed under reduced pressure at 40 °C and then the residue was co-evaporated with ethanol (3 x 500 mL). The resulting solid was dried for 36 h at 60 °C under

reduced pressure to give 47.6 g disodium salt **8** (yield: quantitative; purity:97.8%; sodium content: 3.1%).

LC/MS (Method 3): R_t = 3.00 min, m/z = 1471.6 ($M+H^+$).

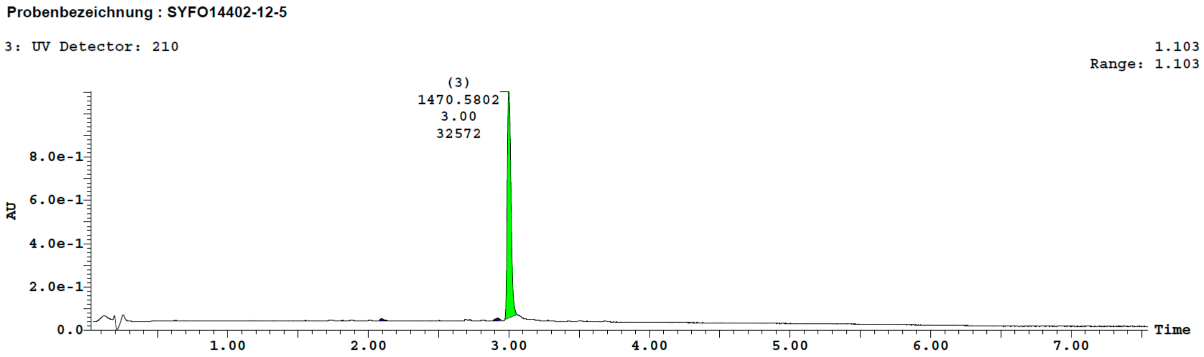

#	Spectrum	RT	Area%	Pos Neg UV	Mass Found
1	---	2.09	0.85	472, 221	
2	---	2.92	1.32	772, 222	
3	---	3.00	97.83	1472, 252	1470.5802

^{1}H NMR (700 MHz, DMSO-d_6) δ ppm 11.3 (br s, 1 H), 9.81 - 9.97 (m, 1 H), 9.35 (br s, 1 H), 8.74 - 8.92 (m, 3 H), 8.27 (br d, *J* = 8.48 Hz, 1 H), 8.16 - 8.23 (m, 1 H), 8.08 - 8.15 (m, 1 H), 8.02 (br d, *J* = 8.2 Hz, 1 H), 7.82 - 7.92 (m, 2 H), 7.71 (br t, *J* = 7.3 Hz, 1 H), 7.59 (s, 1 H), 7.50 (br s, 1 H), 7.20 - 7.39 (m, 6 H), 7.17 (br d, *J* = 7.2 Hz, 1 H), 7.05 (br d, *J* = 6.0 Hz, 2 H), 6.93 (br s, 1 H), 6.64 (br d, *J* = 6.4 Hz, 1 H), 5.46 (br s, 2 H), 5.28 - 5.38 (m, 2 H), 5.16 (br d, *J* = 8.2 Hz, 1 H), 4.96 (br s, 1 H), 4.79 - 4.86 (m, 1 H), 3.95 - 4.03 (m, 1 H), 3.80 - 3.91 (m, 2 H), 3.61 - 3.68 (m, 1 H), 3.39 - 3.55 (m, 12 H), 3.13 - 3.26 (m, 4 H), 2.93 (br d, *J* = 5.5 Hz, 2 H), 2.66 (br dd, *J* = 14.4, 8.9 Hz, 1 H), 2.56 - 2.61 (m, 1 H), 2.33 - 2.46 (m, 2 H), 2.14 - 2.31 (m, 4 H), 2.05 - 2.13 (m, 1 H), 1.91 - 2.04 (m, 2 H), 1.81 - 1.90 (m, 1 H), 1.28 - 1.41 (m, 5 H), 1.01 (br d, *J* = 5.7 Hz, 1 H), 0.86 - 1.02 (m, 9 H), 0.83 (br t, *J* = 7.2 Hz, 3 H).

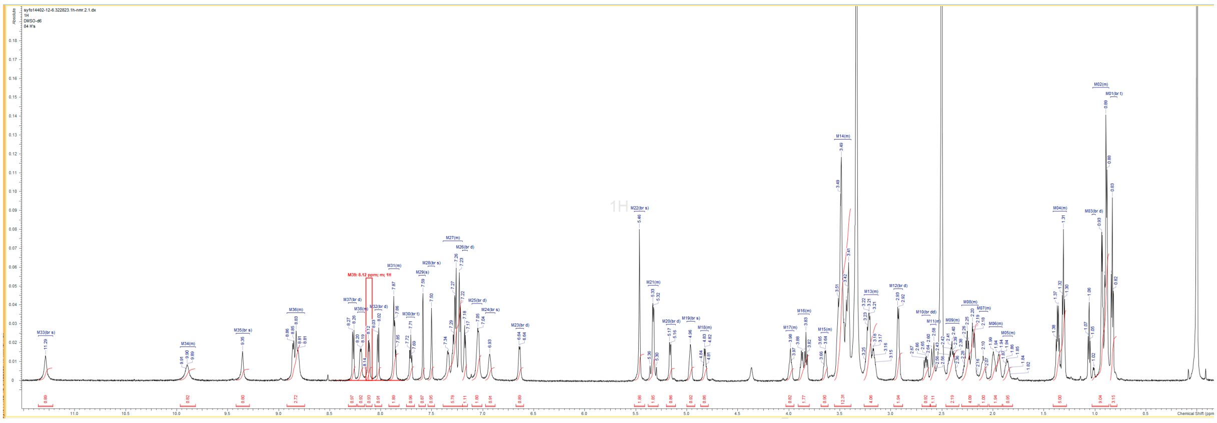

Current Data Parameters
NAME 322824
EXPNO 401
PROCNO 1

F2 - Acquisition Parameters
Date_ 20210705
Time 16.43 h
INSTRUM Avance
PROBHD Z150331_0002 (
PULPROG zgpg
TD 65536
SOLVENT DMSO
NS 1024
DS 4
SWH 41666.668 Hz
FIDRES 1.271566 Hz
AQ 0.7864320 sec
RG 101
DW 12.000 usec
DE 18.00 usec
TE 298.0 K
D1 4.00000000 sec
D11 0.03000000 sec
TD0 8
SFO1 176.1081841 MHz
NUC1 13C
P1 12.50 usec
PLW1 78.81600189 W
SFO2 700.3028012 MHz
NUC2 1H
CPDPRG[2 waltz65
PCPD2 65.00 usec
PLW2 0.24657001 W
PLW12 0.00472328 W
PLW13 0.00236731 W

F2 - Processing parameters
SI 65536
SF 176.0906781 MHz
WDW EM
SSB 0
LB 1.00 Hz
GB 0
PC 1.40

[illegible]

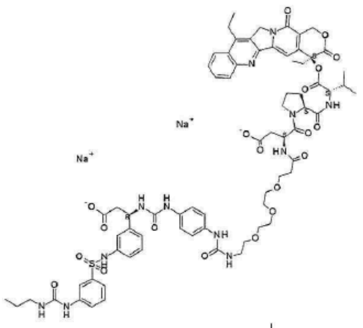

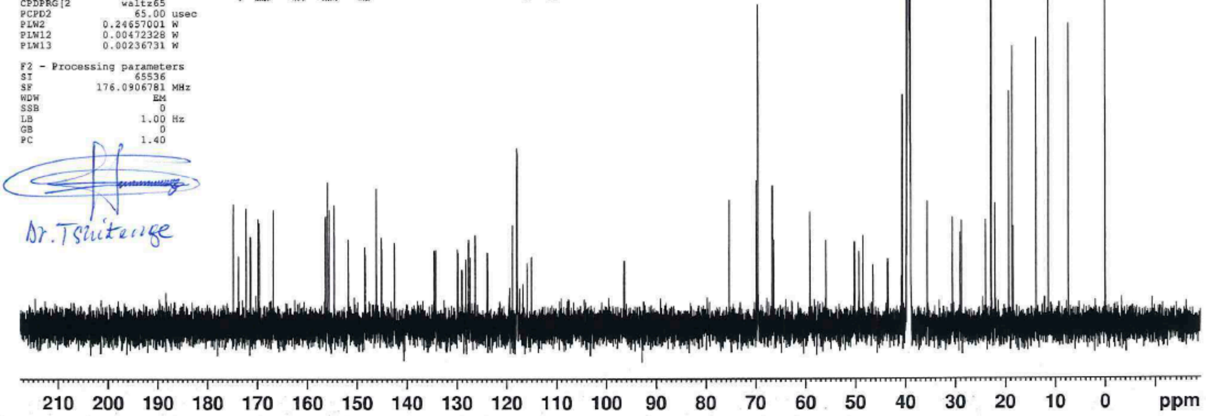

Dr. Tshitenge

CH,CH3-Signals positive ; CH2-Signals negative

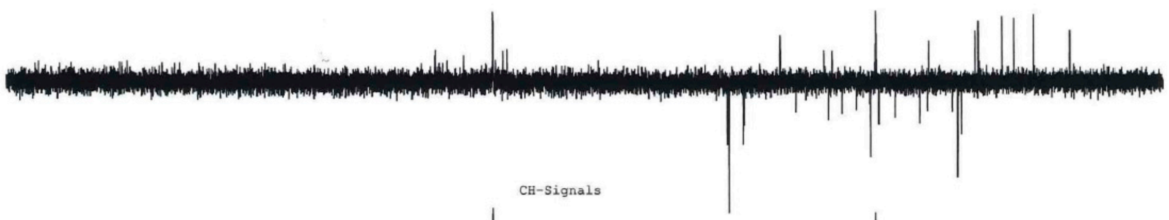

CH-Signals

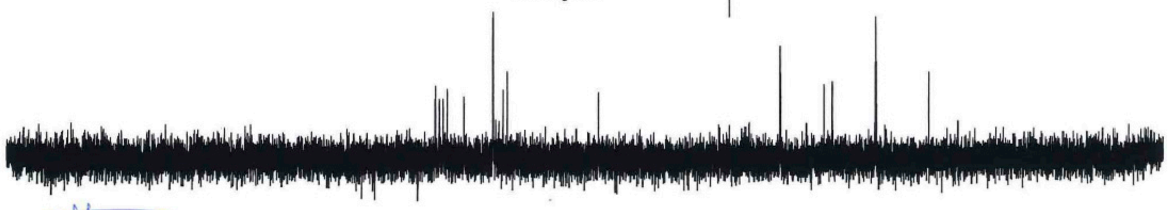

Dr. Tshitenge

SYFO14402-12-6(25.6mg/400μL)

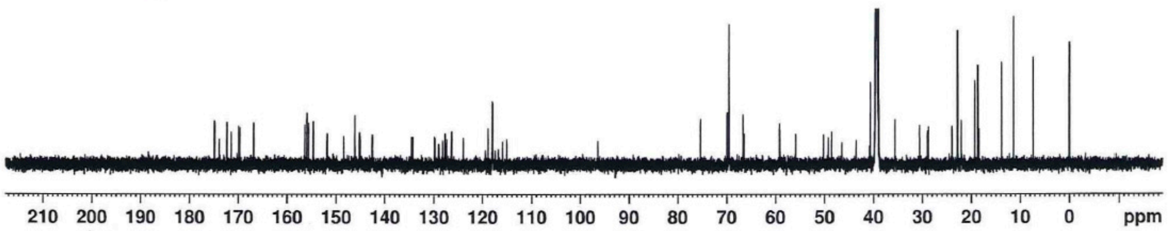

Synthesis of the non-cleavable SMDC epimer 8e1

Disodium (4S)-4,11-diethyl-3,14-dioxo-3,4,12,14-tetrahydro-1H-pyrano[3',4':6,7]indolizino [1,2-b]quinoline-4-yl 1-{(2S)-2-(carboxylatomethyl)-17-[4-({[(1R)-2-carboxylato-1-{3-[({3-[(propylcarbamoyl)amino]phenyl}sulfonyl)amino]phenyl}ethyl]carbamoyl} amino)anilino]-4,17-dioxo-7,10,13-trioxa-3,16-diazaheptadecan-1-oyl}-L-prolyl-D-valinate

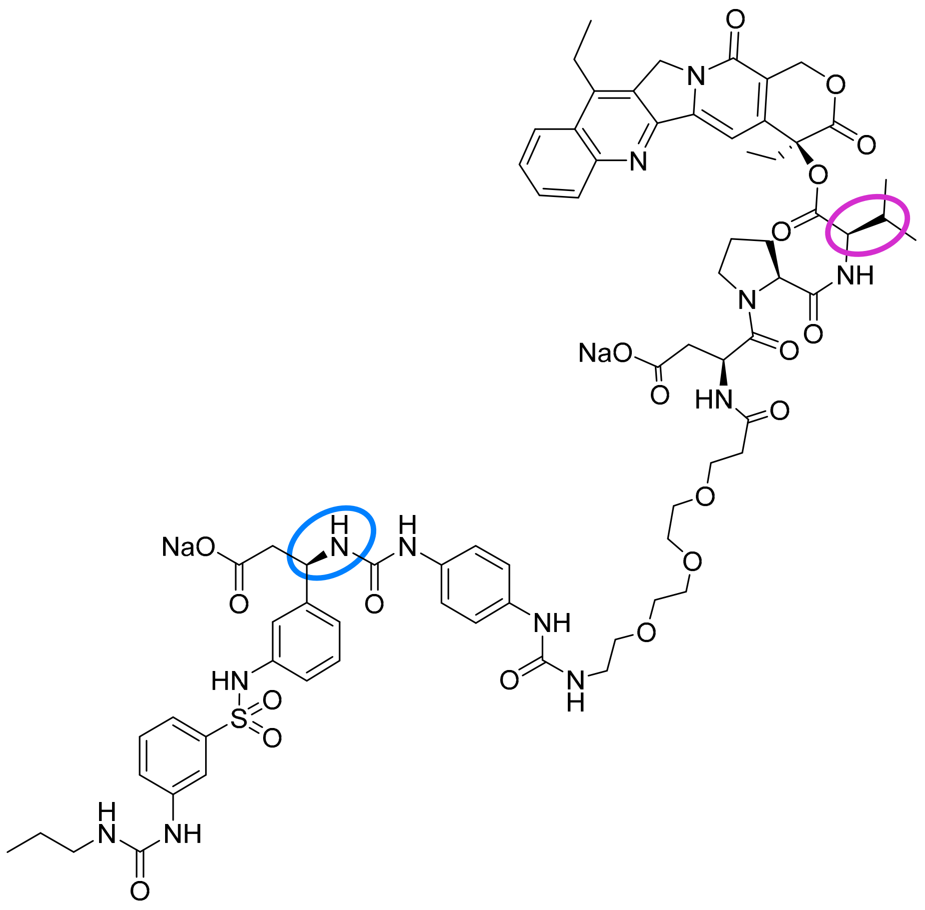

8e1 VIP407 (D-Val / R-IL)

Chemical Formula: $C_{72}H_{84}N_{12}Na_2O_{20}S$

Exact Mass: 1514,5441

Molecular Weight: 1515,5675

In the first step of the synthesis outlined in scheme 1 the opposite enantiomer Boc-D-Valine-N-carboxy-anhydride was employed to acylate the 20-hydroxy group of 7-ethyl camptothecin. All subsequent reaction steps were performed following the same route as described above for **8**. The control SMDC **8e1** was obtained in high purity.

LC/MS (Method 1): Rt = 0.95 min; MS (ESIpos): m/z = 1472 (M - 2Na$^+$ + 2H$^+$ H)$^+$.

Probenbezeichnung : LEE8191-1-2

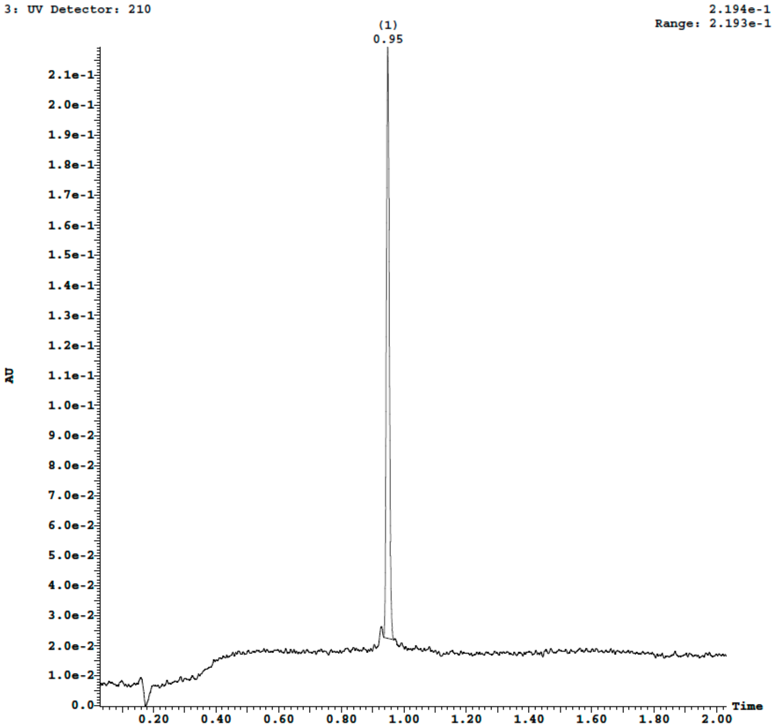

Peak Number	Time	AreaAbs	Area %Total	Mass Found
1	0.95	2149	100.00	

Synthesis of weakly binding epimer 8e2

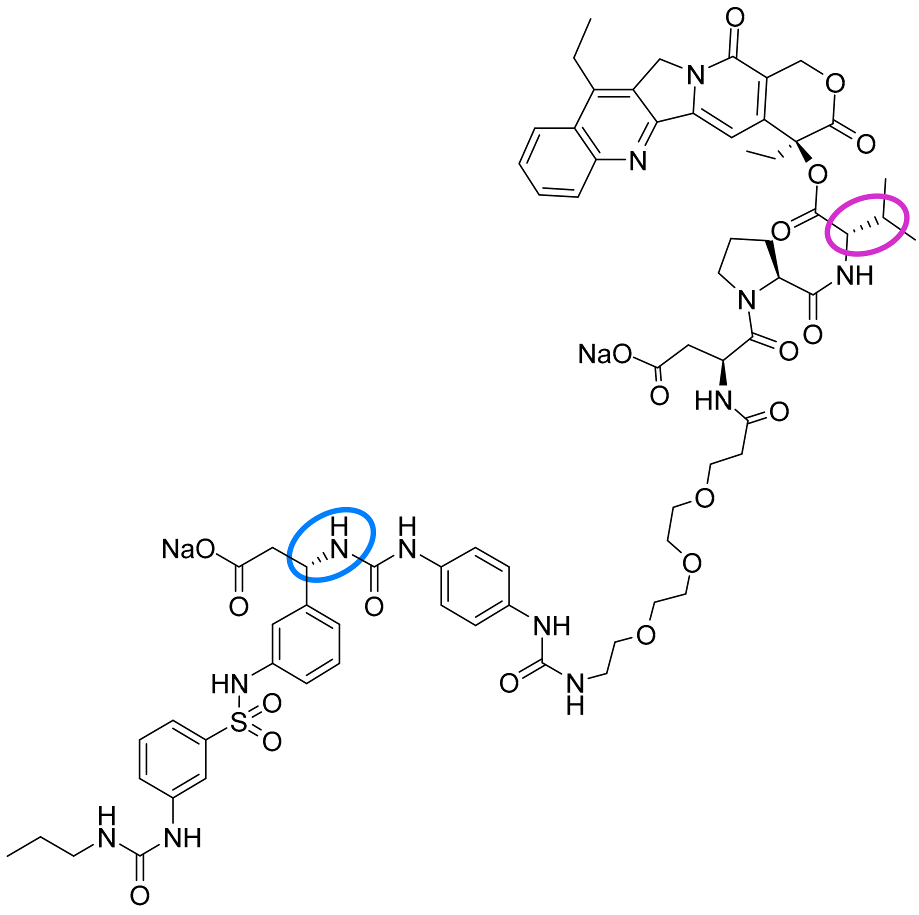

8e2 VIP940 (L-Val / S-IL)

Chemical Formula: $C_{72}H_{84}N_{12}Na_2O_{20}S$
Exact Mass: 1514,5441
Molecular Weight: 1515,5675

a) *Synthesis of activated integrin ligand epimer* ***7e2***

(3S)-3-{[(4-{[(4-nitrophenoxy)carbonyl]amino}phenyl)carbamoyl]amino}-3-{3-[({3-[(propyl carbamoyl)amino]phenyl}sulfonyl)amino]phenyl}propanoic acid (**7 S enantiomer)**

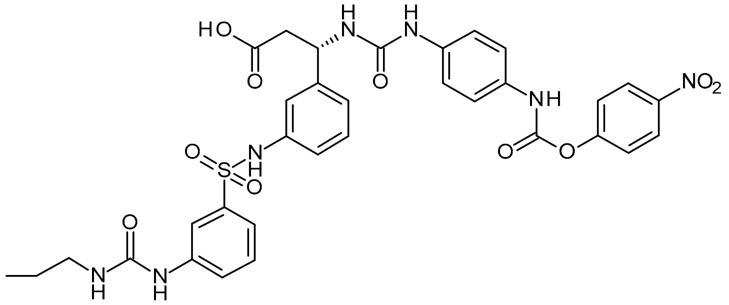

The synthesis of has been performed following the same route as described for its R-enantiomer in WO2020/094471; after chiral separation of the intermediate the opposite enantiomer was employed in subsequent reaction steps.

LC/MS (Method 1): Rt = 0.97 min; MS (ESIpos): m/z = 720 (M+H)+.

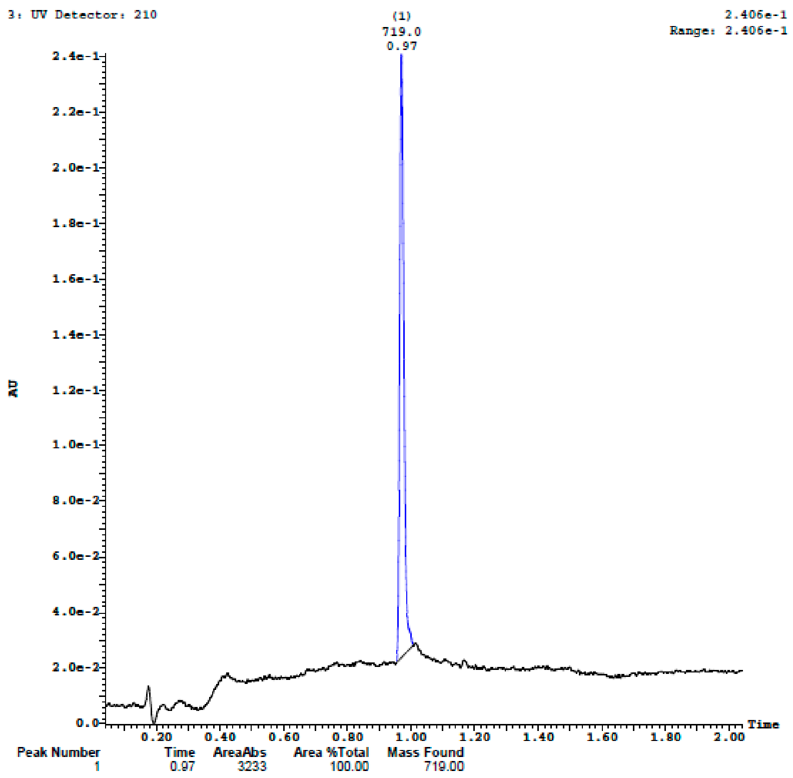

Chiral HPLC: For compound **7e2** an ee > 99% has been determined by chiral supercritical fluid chromatography (SFC) as shown above in table S1 in comparison to its enantiomer **7**.

*b) Synthesis of the weakly binding SMDC epimer **8e2***

Disodium (4S)-4,11-diethyl-3,14-dioxo-3,4,12,14-tetrahydro-1H-pyrano[3',4':6,7] indolizino[1,2-b]quinoline-4-yl1-{(2S)-2-(carboxylatomethyl)-17-[4-({[(1S)-2-carboxylato-1-{3-[({3-[(propylcarbamoyl)amino]phenyl}sulfonyl)amino]phenyl}ethyl]carbamoyl} amino)anilino]-4,17-dioxo-7,10,13-trioxa-3,16-diazaheptadecan-1-oyl}-L-prolyl-L-valinate

The synthesis of **8e2** has been performed in analogy to the synthesis of the epimer **8** as outlined in scheme 1, however using the opposite enantiomer **7e2** instead of **7**. The control SMDC was obtained in high purity.

LC/MS (Method 1): Rt = 0.97 min; MS (ESIpos): m/z = 1471.7 (M - $2Na^+$ + $2H^+$ + H)$^+$.

Probenbezeichnung : LEE8147-3-1

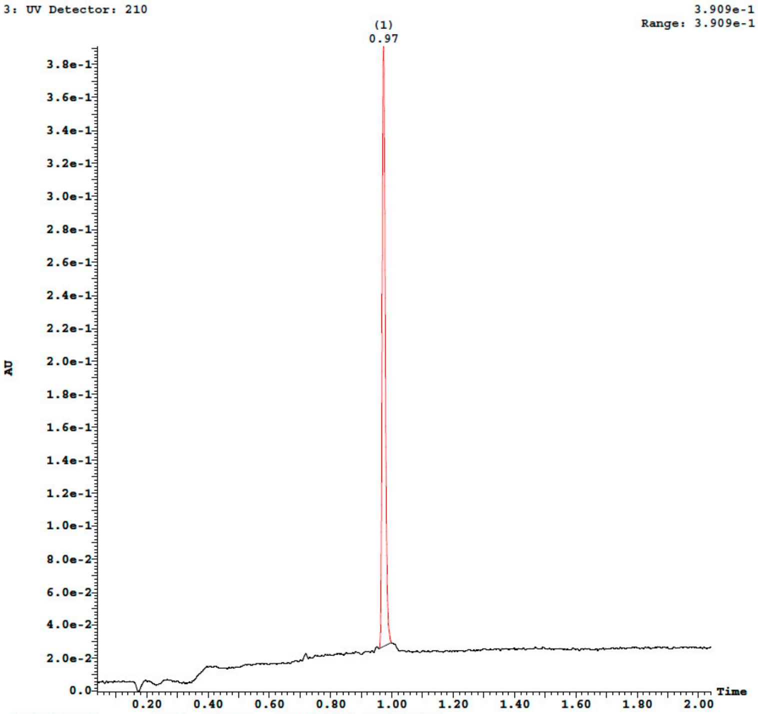

Peak Number	Time	AreaAbs	Area %Total	Mass Found
1	0.97	4303	100.00	

2. Supplementary Tables and figures

a) Supplementary tables

Table S1: Determination of enantiomeric purity of **7** and **7e2**

Sample	Area Ena 1	Area Ena 2	Area% Ena 1	Area% Ena 2	ee% Ena 1	ee% Ena 2
CHRM10484-enantiomeric mix	1088.14	1826.1	37.34%	62.66%	-25.32%	25.32%
CHRM10484-5 enantiomer **7**	8713.75	0	100,00%	0.00%	100.00%	
CHRM10484-6 enantiomer **7e2**	0.00	26300	0,00%	100.00%		100.00%

Table S2: Pharmacokinetics of **8** (VIP236), **8e2** and **8e1** in Female NMRI nu/nu Tumor Bearing Mice Following Administration of a Single IV Dose of 4 mg/kg **8**, **8e2** or **8e1**.

PK parameters	**8** (VIP236)	8e2	8e1
Dose [mg/kg]	4	4	4
CL_{plasma} [L/h/kg]	0.0428	0.111	0.447
V_c [L/kg]	0.0357	0.0483	0.0717
V_{ss} [L/kg]	0.0688	0.0640	0.132
$t_{1/2}$ [h]	0.807	1.30	1.20

Table S3: Summary of treatment data of MA15191 model

Group	n	Treatment	Route	Treatment Days	Dose (mg/kg)	Opt. T/C (%) (day 47)
A	5	PBS	i.v.	d33, 34, 40, 41, 47, 48, 54, 55		-
B	5	VIP236	i.v.	d33, 34, 40, 41, 47, 48, 54, 55	40	17*
C	5	VIP236	i.v..	d33, 40, 47, 54	60	23

**significant compared to control (mixed-effects analysis and Kruskal-Wallis test), p-value=0.015*

b) Supplementary Figures

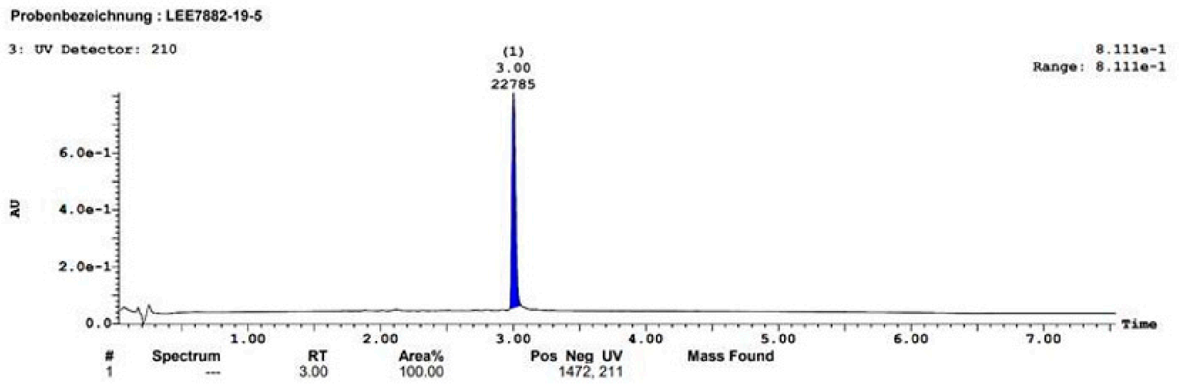

Figure S1: Stability of **8** (VIP236) in PBS buffer: LC/MS after storage for 79d in PBS buffer at 4°C

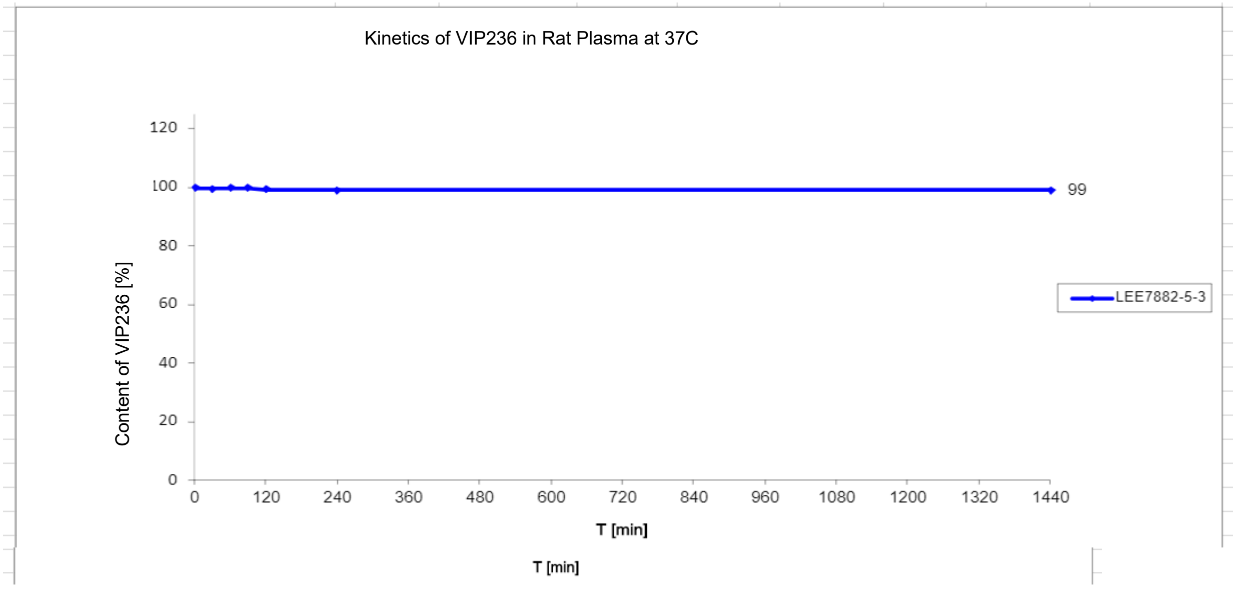

Figure S2: Stability of **8** (VIP236) in rat plasma after incubation for 24h

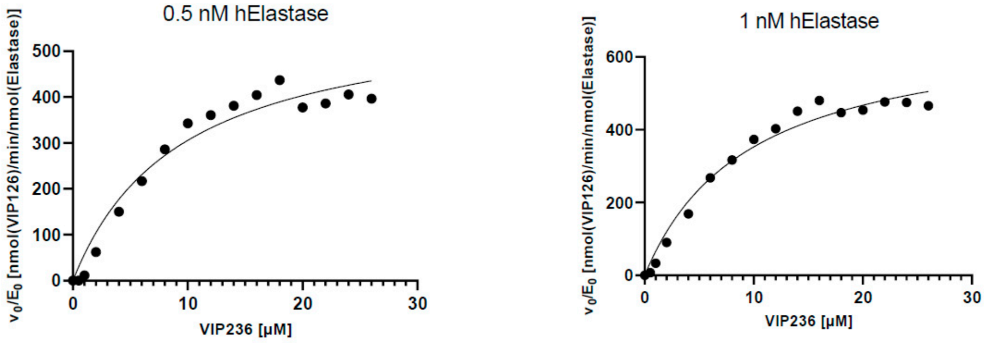

Figure S3: Michaelis-Menten kinetics of **8** (VIP236) in human elastase

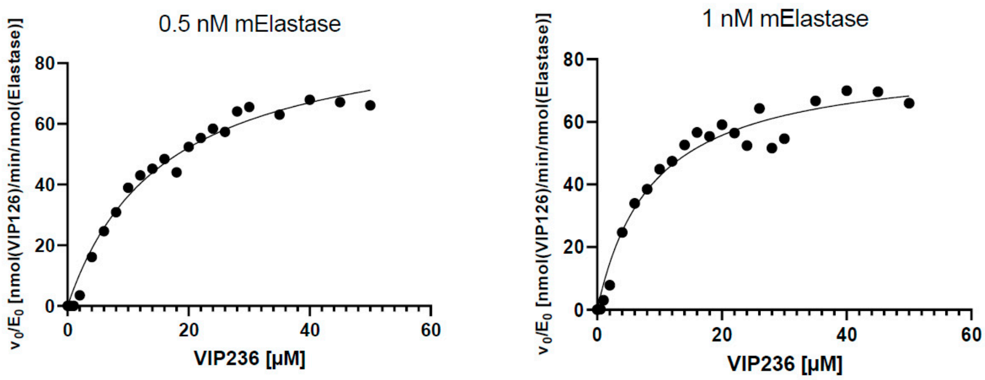

Figure S4: Michaelis-Menten kinetics of **8** (VIP236) in mouse elastase

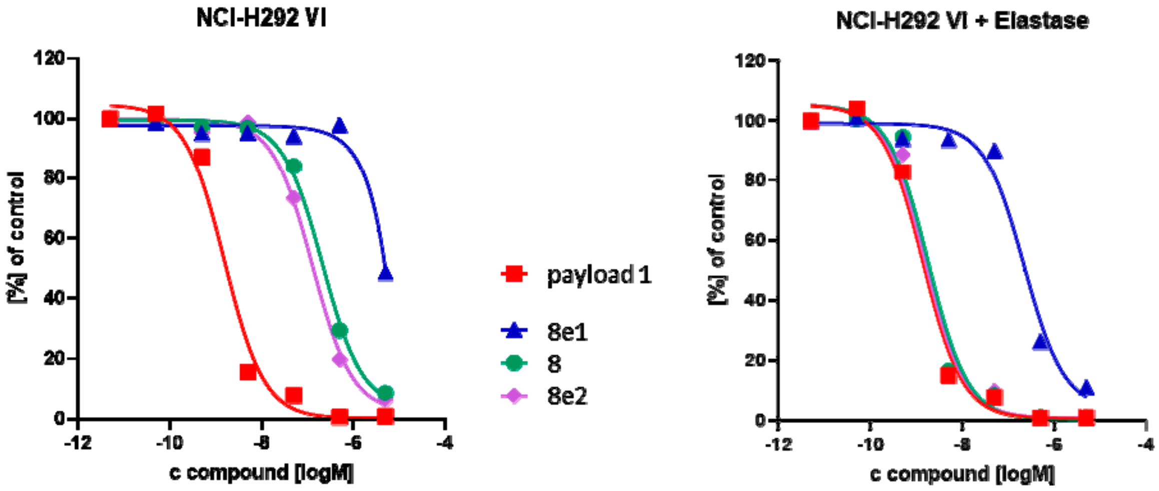

Figure S5. Evaluation of VIP236 and 8e1 and 8e2 in a cytotoxicity assay in the absence or presence of human neutrophil elastase in NCI-H292 cell line.

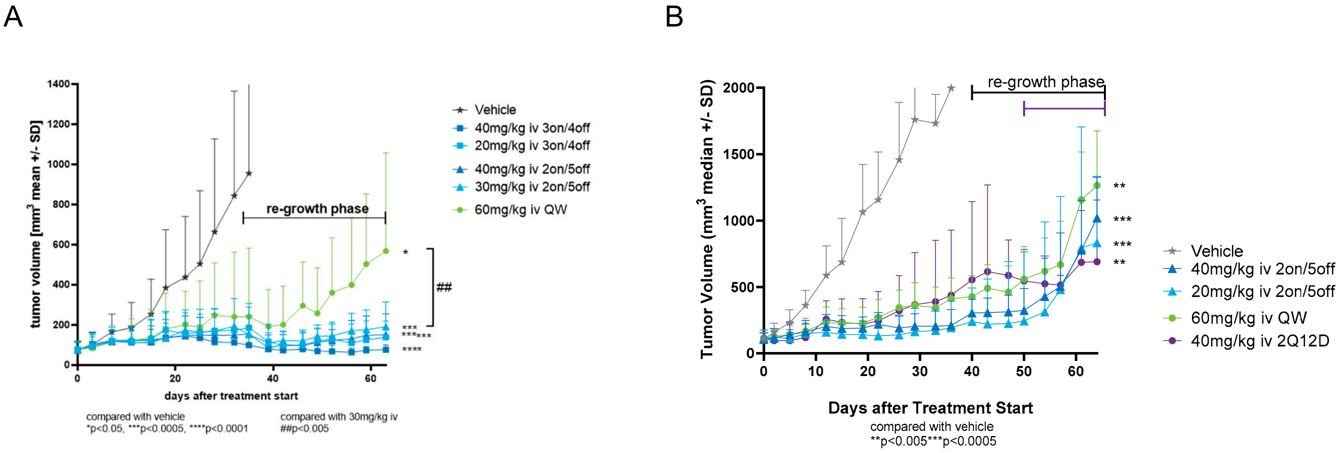

Figure S6: Additional PDX models treated successfully with **8** (VIP236) in monotherapy. **(A)** Growth curves of patient-derived renal cancer RXF 2667 model. All regimes achieved statistically significant tumor growth inhibition. At the end of the re-growth phase partial regression was still observed in the 3on/4off and 2on/5off schedules demonstrating sustained antitumor activity compared with once weekly treatment ($p<0.005$). **(B)** Growth curve of patient-derived breast cancer MAXF BR120 model. Statistically significant tumor growth inhibition was observed at all doses and schedules compared with vehicle as indicated in the graph.

A)

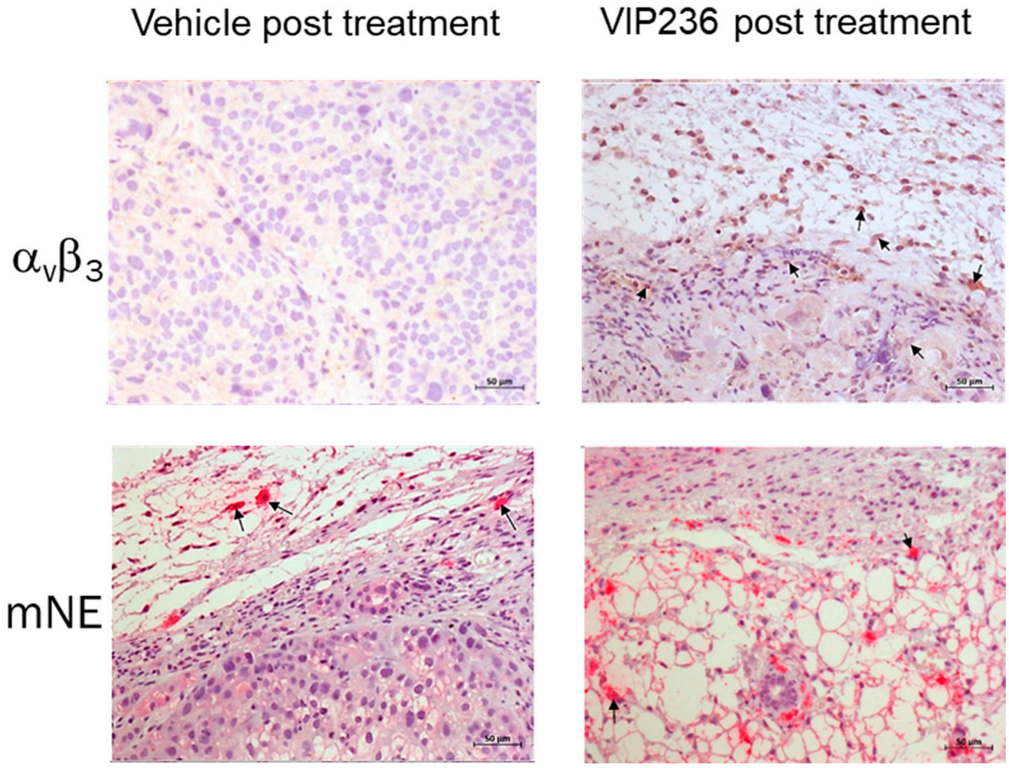

B)

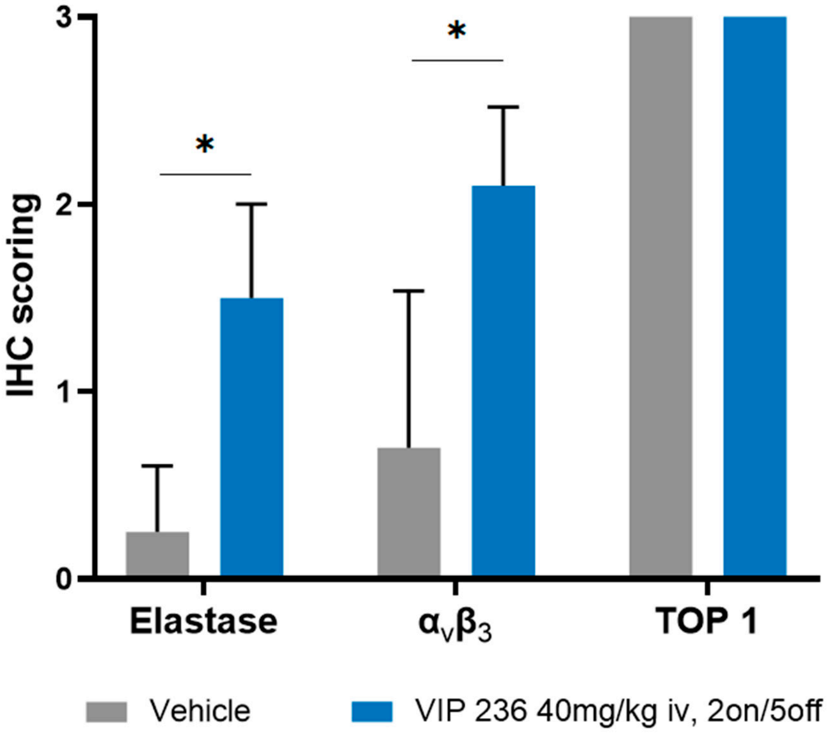

Figure S7: Expression of αvβ3 and TOP1. A) Representative IHC images exhibit increases in αvβ3 and mouse neutrophil elastase (mNE). B) Median of IHC scores displayed comparing

vehicle with treated group (n= 5 animals). Statistical significance shown (unpaired t-test: $p<0.05$)
